# A Case of Type 1 Diabetes Mellitus With Endogenous Insulin Secretory Depletion Confirmed in Two Weeks

**DOI:** 10.7759/cureus.55616

**Published:** 2024-03-06

**Authors:** Hiroki Takizawa, Osamu Ogawa

**Affiliations:** 1 Department of Diabetes and Endocrinology, Kameda Medical Center, Kamogawa-City, JPN; 2 Department of Metabolism and Endocrinology, Juntendo University Graduate School, Tokyo, JPN; 3 Information Management Headquarters, Kameda Medical Center, Kamogawa-City, JPN

**Keywords:** acute-onset type 1 diabetes mellitus, c-peptide, diabetic ketoacidosis, fulminant type 1 diabetes mellitus, type 1 diabetes mellitus

## Abstract

Type 1 diabetes mellitus (T1DM) is manifested as a decrease in endogenous insulin secretion. With this report, we present a case of T1DM where a rapid decline in insulin secretion was observed in a short span of time. A 56-year-old female patient presented with cold-like symptoms with subsequent dry mouth and malaise to the hospital. Three weeks later, she was diagnosed with diabetic ketoacidosis based on the presence of hyperglycemia, metabolic acidosis, and positive ketone bodies. Her serum connecting peptide (CPR) levels substantially decreased (1.31 to 0.19 ng/mL after two weeks) and she was eventually diagnosed with T1DM. We hypothesized that a subtype T1DM with a longer beta cell loss rate than conventional fulminant type 1 diabetes was involved. This subtype showed characteristics of immune checkpoint inhibitor-associated fulminant type 1 diabetes and is suggested to exist among those diagnosed with conventional acute-onset type 1 diabetes. Finally, we recommend that diabetic ketoacidosis of unknown etiology should be investigated for the concurrent presence of T1DM.

## Introduction

Unlike type 2 diabetes mellitus, which is primarily characterized by insulin resistance or insulin deficiency due to genetic or environmental factors, type 1 diabetes mellitus (T1DM) is mainly caused by autoimmune destruction of pancreatic islet β-cells [1]. It is known that there are differences in the incidence rates of T1DM among different racial groups. In populations of white children, including those in Nordic countries, the incidence rate was 36 cases year/100,000 [2], whereas in Japanese children, it was lower at 1.63 cases year/100,000 [3]. T1DM was traditionally considered a disease of children; however, it is now known that besides the initial peak in incidence occurring between ages 10 to 19, there is also a recognized peak in onset occurring after the age of 40 [4].

A consensus report by the American Diabetes Association (ADA) and the European Association for the Study of Diabetes suggests that T1DM should be considered in patients younger than 35 years old, with a BMI of less than 25 kg/m^2^, or in cases where unintentional weight loss or ketoacidosis is present [5]. The ADA guidelines state that islet autoantibodies should be measured to diagnose immune-mediated diabetes and emphasize that the rate of destruction of beta cells by autoimmunity is variable [6]. Additionally, it is known that immune checkpoint inhibitor (ICI)-associated T1DM often presents with negative islet autoantibodies in approximately half of cases, leading to a fulminant onset of T1DM [6]. On the other hand, diabetes mellitus causing diabetic ketoacidosis, characterized by antibody negativity and insulin deficiency, is classified as Idiopathic T1DM [7].

In Japan, other types of T1DM include acute-onset T1DM and latent autoimmune diabetes in adults. Fulminant T1DM (FT1DM) is a subtype of acute-onset T1DM in Japanese patients. FT1DM is characterized by 1) high incidence in adulthood, 2) negativity for islet-associated autoantibodies, 3) rapid decline in insulin secretion within a short period, 4) elevated levels of pancreatic exocrine enzymes in the blood at onset, and 5) diabetic ketoacidosis. FT1DM is estimated to account for approximately 20% of acute-onset T1DM cases [8].

FT1DM describes a disease that results in ketosis or ketoacidosis within one week of the onset of diabetic symptoms [9]. On the other hand, it is said that ICI-associated TIDM has a different onset pattern from conventional T1DM. In ICI-associated T1DM, there appears to be a longer duration of diabetes, with beta cell loss occurring for approximately two weeks longer compared to conventional T1DM [10]. It has been traditionally assumed that FT1DM leads to ketoacidosis within one week; however, in this report, we present the case of a T1DM patient who was never treated with ICI but showed a decline in endogenous insulin secretion within two weeks of onset.

## Case presentation

A 56-year-old female patient presented with symptoms of thirst and malaise. Three weeks prior, she reported symptoms resembling a cold. Subsequently, she performed a urine test using a test strip at home, which revealed a positive finding of glucose. She possessed a past medical history of primary aldosteronism and was being treated with esaxerenone at the time. No previous family history of diabetes existed. On arrival, her vital signs were within normal limits. Hemoglobin A1c (HbA1c) increased from 5.7% (six months before) to 8.3% while blood glucose level, serum connecting peptide (CPR), pH, and total ketone bodies were detected at 425 mg/dL, 1.31 ng/mL, 7.28, and 4,858 μmol/L, respectively. Thus, the patient was diagnosed with diabetic ketoacidosis (Table 1).

**Table 1:** Blood test results on admission Abbreviations: ALT, alanine aminotransferase; AST, aspartate aminotransferase; CPR, connecting peptide immunoreactivity; GAD, glutamic acid decarboxylase

Tested Parameters	Patient’s serology testing	Reference Range
Hemoglobin, g/dL	16.6	12-15
Red blood cell count, ×10^12^/L	5.63	3.8-5.2
White blood cell count, ×10^9^/L	740	4-11
Platelets, ×10^9^/L	235	150-400
AST, U/L	11	0-35
ALT, U/L	15	0-40
Amylase, U/L	76	39-134
Lipase, U/L	125	17-57
Creatine Phosphokinase (CPK), U/L	97	45-163
Creatinine, mg/dL	0.61	0.47-0.79
Urea, mg/dL	17	8-22
Sodium, mEq/L	131	136-147
Potassium, mEq/L	4.6	3.6-5.0
Calcium, mg/dL	10.3	8.5-10.2
Glucose, mg/dL	425	70-109
HbA1c, %	8.3	4.6-6.2
Total ketone bodies, μmol/L	4858	0-131
Acetoacetic acid, μmol/L	854	0-55
3-hydroxybutyric acid, μmol/L	4004	0-85
pH	7.28	7.35-7.45
HCO_3_^-^, mmol/L	19.5	23-27
CPR, ng/mL	1.3	0.61-2.09
anti-GAD antibodies, U/mL	Negative	0-4.9
Insulin autoantibodies, U/mL	Negative	0-0.4
Islet Antigen 2 Antibody, U/mL	Negative	0-0.6

**Figure 1:**
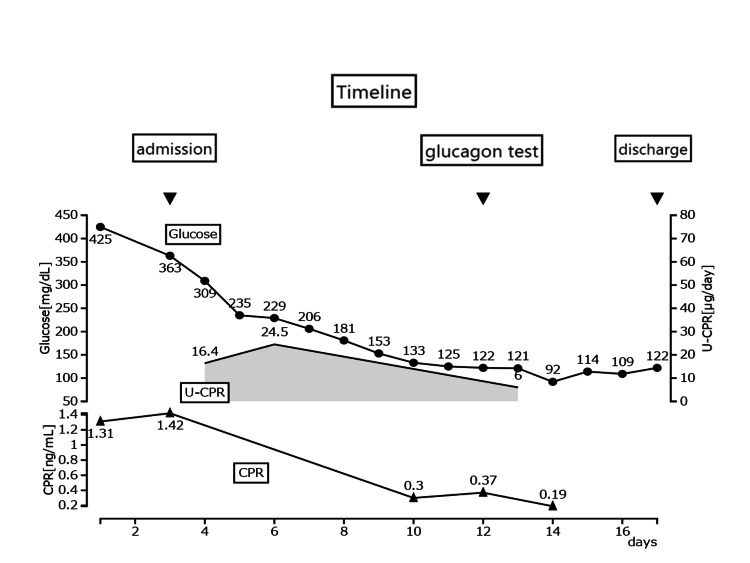
Course of treatment She was admitted to the hospital on the third day following the initial visit. CPR levels decreased progressively, ultimately reaching a fasting CPR value of 0.19 ng/mL.

She could not be admitted to the hospital on the first visit and was given fluids and intravenous insulin in an outpatient setting. After administration of 2000 ml saline solution and 10 units of human insulin, pH and blood glucose improved to 7.33 and 70 mg/dL, respectively. Insulin therapy was introduced, and she was admitted to the hospital after three days. Multiple dose insulin therapy improved her general condition, but serum CPR decreased to 0.19 ng/mL over time. Anti-glutamic acid decarboxylase antibody, islet tyrosine phosphatase 2 antibody, and anti-insulin antibodies were absent. A glucagon test on the 12th day confirmed a ΔCPR of 0.4 ng/mL. She was discharged on the 15th day of hospitalization as normal blood glucose was restored with 7-7-7 units of insulin Aspart and 15 units of insulin Degludeg (Figure 1).

## Discussion

Our case suggests the existence of a subtype of Subfulminant T1DM in which endogenous insulin secretion is depleted in about two weeks. We found that repeated measurements of serum CPR are useful for diagnosis, even for a short period of time. In addition, we suggest that this subtype of diabetes mellitus, termed subacute T1DM, manifests as a depletion in endogenous insulin secretion after two weeks of diagnosis. In our case, the patient, who presented in adulthood, was diagnosed with diabetic ketoacidosis, HbA1c <8.7 %, elevated lipase, and negative islet-associated antibodies at the first visit. These findings are consistent with FT1DM. However, the endogenous insulin secretory capacity was not completely depleted, and the diagnostic criteria for FT1DM were not met at the time. Similarly, the diagnostic criteria for acute onset T1DM were also not met. However, hyposecretion of endogenous insulin was observed after 15 days suggesting that endogenous insulin secretion decreased about two weeks after the onset of hyperglycemic symptoms. It is possible that if she had come to the hospital after five days, FT1DM may have been diagnosed. Therefore, we diagnosed FT1DM where the endogenous hyposecretion of insulin could be observed.

Recently, a type of ICI-related FT1DM has been reported in which insulin hyposecretion is relatively slower than in conventional FT1DM [10]. Hereon, termed as subfulminant T1DM, it manifests in FT1DM patients with a decrease in endogenous insulin secretion over a period of two to three weeks and can be considered subFT1DM. One case of ICI--ICI-associated FT1DM has been reported where serum CPR level decreased from normal to below the measurement sensitivity in about two weeks [11]. In another case report, FT1DM with hyperglycemia was confirmed three weeks before diagnosis [12]. Furthermore, the rate of beta cell loss in the current patient was comparable to that in patients with ICI-associated FT1DM (as observed by a decrease in CPR in two weeks) although she had never received ICI. In addition, HbA1c was higher than that of conventional FT1DM as well as ICI-related FT1DM [13]. This assumption is further supported by the fact that insulin secretion decreased at a rate similar to that of ICI-associated FT1DM. A subtype of FT1DM, called subFT1DM with reduced endogenous insulin secretion over a period of two to three weeks, may exist even in the absence of ICI-associated FT1DM. In addition, the depletion of endogenous insulin secretion after a certain period, although high Hb1Ac was observed from the initial diagnosis, may suggest that the decrease in beta cells is not linear but quadratic. Further studies are needed to confirm this scenario.

To understand the relationship between the mode of endogenous insulin secretion depletion and human leukocyte antigen (HLA), a few reports could be identified that showed that HLA-A24 promoted beta cell destruction [14]. Other high-risk HLAs for immune checkpoint-related FT1DM include HLA-DR4, which is known to be possessed by as many as 76% of individuals [15]. Additionally, HLA-DR4 has been reported to be as high as 44.7% in Japanese patients with FT1DM [16]. However, we could not measure HLA. Evaluation of HLA in similar cases may advance our understanding of the disease progression. Similarly, because T1DM with ketoacidosis may cause endogenous insulin depletion on a daily basis, serum CPR levels may be useful for diagnosis, even for short periods of time. In our case, the serum CPR was relatively low on admission and did not meet the diagnostic criteria of acute onset T1DM. However, the serum CPR decreased substantially in two weeks, suggestive of depletion of endogenous insulin secretion. Reports of hyperinsulinemia leading to hypoglycemia before the onset of FT1DM exist [17]. Similarly, primary hyperinsulinemia has been shown to occur in a mouse model of T1DM [18]. The above reports may explain the preservation of endogenous insulin secretion in our patient during initial diagnosis. In addition, as the serum CPR changes dynamically over a period of several days even in T1DM, the possibility of T1DM should not be excluded based on the serum CPR levels at the initial diagnosis, especially in cases of diabetic ketoacidosis with unknown triggers.

## Conclusions

The category of unexplained ketoacidosis encompasses antibody-negative fulminant type 1 diabetes. There exists a relatively subfluminat type, and assessing repeated insulin secretion capacity, even over a short duration, proves to be valuable. While the classification of disease types in type 1 diabetes is important, it is also crucial to evaluate patients' endogenous insulin secretion. This facilitates early recommendations for continuous glucose monitoring and insulin pump therapy.
